# Effects of Cadmium Stress on Root Exudates and Soil Rhizosphere Microorganisms of Rice (*Oryza sativa* L.) and Its Ecological Regulatory Mechanisms

**DOI:** 10.3390/plants14111695

**Published:** 2025-06-01

**Authors:** Siqi Lin, Qing He, Mingxia Zhang, Yingyi Huang, Huahong Liu, Qi’er Mu, Sheng Wang, Jinfang Nie

**Affiliations:** 1 College of Chemical and Bioengineering, Guilin University of Technology, Guilin 541004, China; 13799575647@163.com (S.L.); 1020200733@glut.edu.cn (Q.H.); huangyingyi2021@163.com (Y.H.); liuhuahong1999@126.com (H.L.); 15848145443@163.com (Q.M.); 2 Department of Ecology, College of Life Science and Technology, Jinan University, Guangzhou 510632, China; zhangmingxia@stu2022.jnu.edu.cn

**Keywords:** Cd stress, rice, root exudates, soil rhizosphere microorganisms, ecological regulatory mechanisms

## Abstract

Rice, one of the global staple food crops, is significantly affected in its growth by cadmium (Cd) contamination in soil. This study comprehensively investigated the impact of Cd stress on the root exudates and rhizospheric soil microorganisms of rice through non-targeted metabolomics and high-throughput 16S rRNA sequencing technologies, as well as the ecological regulatory mechanisms between them. Root exudates reflect proactive plant defenses and enhance these capabilities by attracting beneficial microorganisms, which play a pivotal role in plant detoxification. There were significant changes in root exudates under Cd stress, their chelation and rejection of Cd ions diminished the bioavailability within the plant system, thereby mitigating the phytotoxic effects of heavy metal stress and safeguarding the overall health of plants. Moreover, Proteobacteria (*Lysobacter*, *Pseudaminobacter*, and *Sphingomonas*) were recruited by the root exudates from rice as potential participants in plant tolerance and detoxification processes. These findings offer novel insights into the ecological adaptability mechanisms of rice under heavy metal stress and provide potential biomarkers and microbial resources for agricultural environmental regulation.

## 1. Introduction

Rice, a staple food crop globally, has been pivotal in ensuring a consistent increase in agricultural productivity worldwide [1]. The rapid industrialization, however, has unfortunately triggered the concerning contamination of rice paddy soils with heavy metals [2]. This environmental trend not only threatens the quality of these vital agricultural lands, but also poses a significant risk to the food chain and public health. Cadmium (Cd), a highly toxic non-essential trace metal, has emerged as an increasingly prominent contaminant in China’s agricultural soils, with southern regions particularly at risk [3]. When rice is planted in Cd-contaminated fields, excessive uptake of Cd by the roots, stems, leaves, and grains results in stunted growth and the reduction of crop yields, even posing significant threats to the integrity and security of rice [4]. Undoubtedly, numerous studies have also shown that rice is not entirely defenseless against Cd contamination. When subjected to a specific degree of Cd stress, rice can activate its inherent defense mechanisms to reduce the accumulation of Cd, thereby demonstrating a level of adaptability to such environmental challenges [5].

Root exudates, which are complex mixtures of plant-derived metabolites secreted into the rhizosphere, represent a vital detoxification mechanism that has evolved in response to environments contaminated with toxic metals [6]. These secondary metabolites can serve as metal precipitators, antioxidants, or metal chelators to alleviate the detrimental impacts of harmful metals, thereby enhancing metal stress tolerance in plants [7]. For instance, root exudates, such as malic and citric acids, act as metal precipitators to stabilize toxic metals in the vicinity of roots, resulting in an enhanced accumulation of chromium in the rhizospheres of *Solanum nigrum* and *Parthenium hysterophorus* [8]. A class of phenolic compounds represents a vital category of aromatic secondary metabolites in plants, exhibiting redox properties that enable them to function as potential antioxidants during periods of stress [9,10]. Manquían-Cerda et al. demonstrated that Cd exposure resulted in a rapid increase in the concentration of chlorogenic acid, the primary phenolic compound within blueberry plantlets. Their results showed a negative correlation between phenolic compound levels and oxidative damage, suggesting these compounds may effectively reduce oxidative stress by scavenging free radicals [11]. Accumulation of anthocyanins in the foliage of *Zea mays* L. was observed when it was subjected to zinc stress, and cyanidin could moderately reduce the detrimental effects of metal toxicity by forming a cyanidin–Zn complex [12]. In addition, in Pb-stressed tomato seedlings, jasmonic acid could enhance the levels of metal-chelating substances, such as thiols, metallothioneins, and phytochelatins [13]. This suggests that secondary metabolites acting as metal chelators constitute an important strategy for combating heavy metal stress.

Although root exudates play a crucial role in metal detoxification through these three mechanisms, each has its inherent limitations. For instance, the precipitation of excess metals may result in the reduction of essential nutrient bioavailability [14]. The efficacy of antioxidant mechanisms is contingent upon the plant species, the specific heavy metal, and its concentration [15], and the effectiveness of metal chelation might be hindered in particular metals or at low pH conditions [6,16]. In this context, the recruitment of beneficial microorganisms by root exudates emerges as a complementary and robust approach to alleviate Cd stress. It has been reported that root exudates possess the capacity to augment the biotic macro-aggregation of soils and foster intricate plant–microbe interactions [17]. Moreover, the capacity of microorganisms to evolve intricate resistance strategies against the detrimental impacts of toxic heavy metal ions reflects their extraordinary adaptability [18]. Root exudates, such as triterpenes and flavonoids, could significantly influence the makeup of microbial communities in the vicinity of the root [19]. Diverse microbial communities rely on organic acids, sugars, and amino acids as a rich source of nutrients [20]. Additionally, the incorporation of exudate compounds from rice, both separately and in combination, significantly reshaped the composition of microorganisms throughout the root system [19]. Hence, this study focuses on examining the hypothesis that plants might recompose the composition of soil microbial communities through the modulation of their root exudates when subjected to Cd stress.

Root exudates mediate the complex interactions between plants and soil microbial communities, which are essential in structuring the composition of soil microbial communities [21]. It has been demonstrated in numerous studies that root exudates not only recruit Cd-resistant microorganisms, but also regulate the microorganisms within the rhizosphere, thereby enhancing plant adaptation to environmental changes [22]. *Bacillus*, *Burkholderia*, *Arthrobacter*, *Sphingosphinomonas*, and *Streptomyces* are known to fix, transform, and adsorb Cd, potentially diminishing its accumulation in rice crops [23]. The enrichment of these microorganisms under Cd-contaminated conditions is exceedingly advantageous for resisting Cd stress. *B. vietnamensis 151-6* and *B. marisflavi 151-25*, isolated from Cd-contaminated arable soils, displayed notable differences in their resistance to and uptake of Cd [24]. Meanwhile, it has been demonstrated that plant growth-promoting bacteria can modify metal availability through various mechanisms, including the production of biosurfactants, altering soil pH, and driving redox reactions. Additionally, these bacteria can help mitigate the toxicity of heavy metals by employing reactive oxygen species (ROS) neutralization systems, such as peroxidases and catalases [25]. Nevertheless, there is a paucity of studies that have concentrated on the comprehensive investigation of root exudates to remodel microbial communities and fortify the resilience of rice to Cd stress. Consequently, the exploration of the complex physiological and metabolic changes induced by Cd in crops, as well as the dynamic patterns of soil microbial community structure and the intricate regulatory mechanisms between the root exudates released under Cd stress and rhizosphere microorganisms, has become a pivotal research subject.

In this study, non-targeted metabolomics technology was used to investigate the alterations in root exudates of rice in response to varying Cd concentrations, thereby facilitating a more profound comprehension of the physiological and metabolic adaptations in plants subjected to Cd stress. The high-throughput sequencing technology was employed to investigate the impact of varying Cd concentrations on soil microbial communities. Meanwhile, Pearson correlation analysis was conducted to reveal the impact of root exudates on microbial communities, showing the regulatory role in plant–microbe interactions. This study provides a novel perspective for a comprehensive and in-depth understanding of the response mechanisms between root exudates and microbial communities in rice under Cd stress.

## 2. Results

### 2.1. Changes in the Cadmium Content of Rice Tissues Under Cd Stress

**Figure 1:**
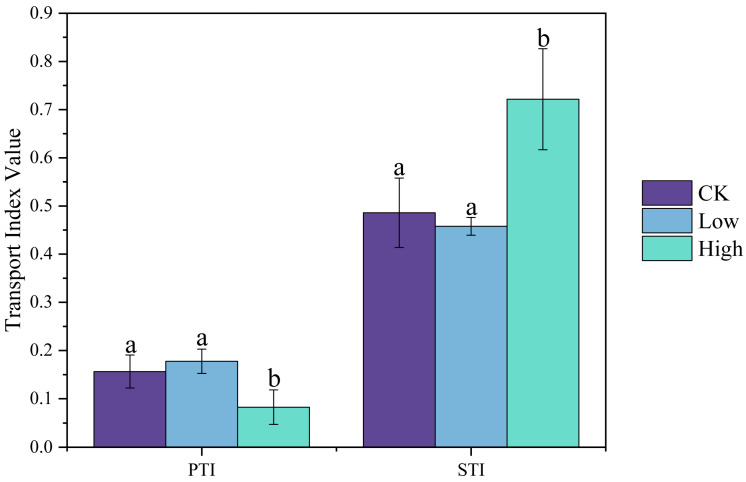
Comparative analysis of cadmium (Cd) translocation indices in rice plants under control and Cd-contaminated conditions. The primary transport index (PTI) represents Cd movement from roots to stems, and the secondary transport index (STI) indicates Cd translocation from stems to leaves. The treatment groups include the control (CK, 0 mg kg^−1^), low Cd concentration (Low, 2 mg kg^−1^), and high Cd concentration (High, 10 mg kg^−1^). Different lowercase letters (a, b) above the bars indicate statistically significant differences between the groups (ANOVA, Tukey’s test, *p* < 0.05). Bars represent the average of three replicates, and error bars indicate the standard deviations.

Transport coefficients were utilized to assess the capability of rice to accumulate and transport heavy metals [26]. In order to comprehensively explore the migration capacity of rice tissues under different Cd contents, the primary transport index (PTI) and the secondary transport index (STI) were used to assess the extent of Cd migration in rice species [27,28]. As depicted in Figure 1, the PTIs of Cd were 0.156, 0.178, and 0.083 in the CK group, Low group, and High group, respectively, and the STIs were 0.486, 0.458, and 0.722.

Comparatively, our focus is on the PTI, which is crucial for understanding the efficiency of Cd translocation from roots to stems in rice, a key aspect of elucidating the internal migration mechanisms of Cd within the rice plant. It can be observed that rice employs distinct strategies to facilitate adaptation to varying levels of Cd stress. With increasing Cd stress, the capacity to translocate Cd from the root to the stem diminishes, leading to a greater accumulation in the root (Table S1). This finding is in close alignment with those of previous research studies in this field [7]. Conversely, the ability for its translocation from the stem to the leaves is enhanced, which may be attributed to the considerable deposition of Cd in the stem, forcing transport proteins to shuttle a large amount of Cd to the leaves. In leaf tissues, Cd is sequestered into extracellular or subcellular compartments [29].

### 2.2. Effect of Cd Stress on Root Exudates of Rice

**Figure 2:**
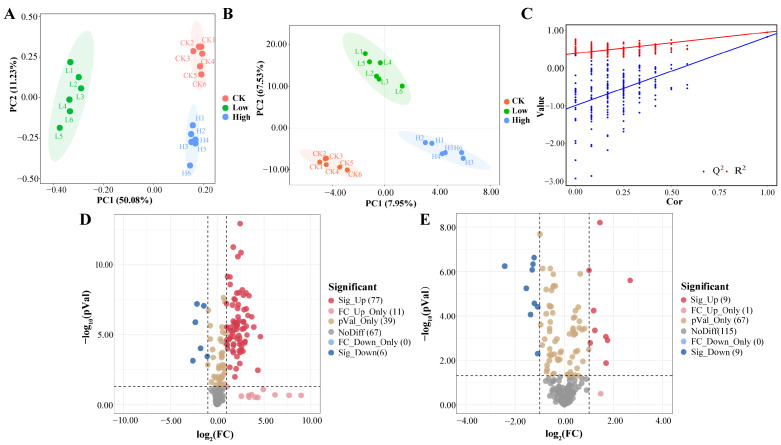
Rice root exudates: (**A**) principal component analysis (PCA), (**B**) partial least squares discriminant analysis (PLS-DA), and (**C**) 200 iterations of cross-validation. Differential root exudates (VIP > 1, *p* < 0.05, FC ≤ 0.5 or FC ≥ 2) in the (**D**) low- and (**E**) high-treatment groups compared to the control group. The treatment groups include the control (0 mg kg^−1^), low Cd concentration (2 mg kg^−1^), and high Cd concentration (10 mg kg^−1^).

**Figure 3:**
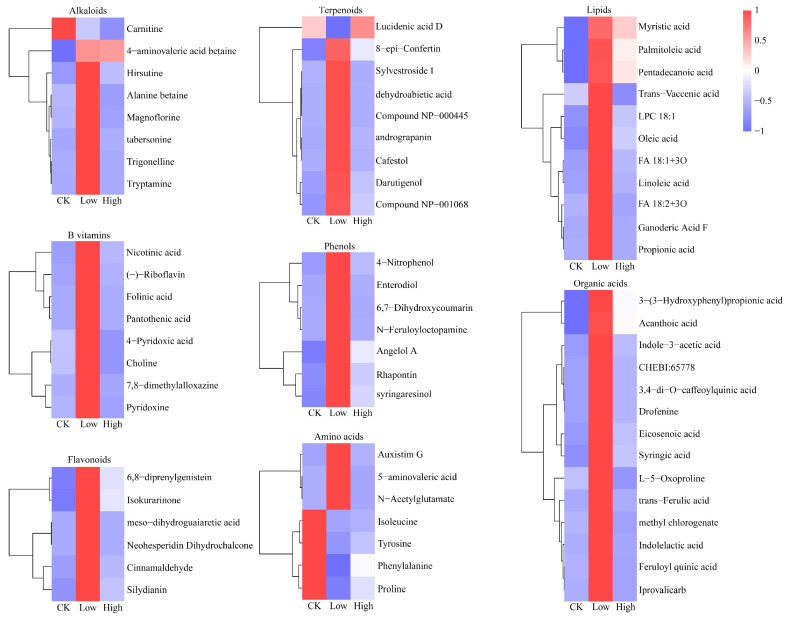
Heatmap analysis of the identified characteristic differential metabolites in rice root tissues (*p* < 0.05). The treatment groups include the control (0 mg kg^−1^), low Cd concentration (2 mg kg^−1^), and high Cd concentration (10 mg kg^−1^). The red and blue blocks indicate significant upregulation and downregulation, respectively.

In this paper, we analyzed root exudates of each treatment group using the LC-QTOF/MS method. A total of 199 metabolites were detected across both positive and negative ion modes. Principal component analysis (PCA) was utilized to analyze the metabolite data to exhibit the similarities and differences between the different treatment groups. As shown in Figure 2A, the first two principal factors collectively accounted for 50.08% and 11.23% of the total variance, respectively. The close clustering of biological replicate samples indicated that the analytical method exhibited reliable reproducibility. The metabolic profiles of rice root exudates from the different treatment groups exhibited notable discrepancies, indicating that Cd stress had a pronounced impact on the metabolism of root exudates. To delineate the metabolic differences more clearly, a cross-validation analysis involving 200 permutation tests was conducted to assess model stability and avoid overfitting concerns. The R^2^ values exceeded their corresponding Q^2^ values for all the samples, confirming that the model was not overfitted and exhibited good stability (Figure 2B,C). In order to more precisely evaluate the differences in chemical properties between the treatment groups, 91 differential metabolites were screened out based on the criterion of *p* < 0.05 combined with VIP > 1 (Table S2). These metabolites were classified as organic acids (14), lipids (11), terpenoids (9), alkaloids (8), vitamin B substances (8), amino acids (7), flavonoids (6), phenols (7), glycosides (4) and other compounds (17) (Figure S2A). The volcano plot combined statistical significance with the scale of change to quickly and visually identify metabolites that had significantly changed. With the fold change criteria (FC ≥ 2 and FC ≤ 0.5), 83 and 18 differential metabolites were screened in the Low/CK treatment group and the High/CK treatment group, respectively. Among them, 77 metabolites were upregulated and 6 metabolites were downregulated in the Low/CK treatment group, while 9 metabolites were upregulated and 9 metabolites were downregulated in the High/CK treatment group (Figure 2D,E). To better visualize the unique and shared metabolic features of root exudates under different Cd treatments, Venn diagram analysis was performed (Figure S2B). The results revealed that the CK, Low, and High groups contained 25, 30, and 51 unique metabolites, respectively, while 37 metabolites were shared across all three groups. The higher number of unique metabolites in the High Cd group suggests that rice roots actively modulate their exudation profile under severe Cd stress, potentially as part of a defense response. Notably, the number of metabolites shared between the groups was lower than the number of unique metabolites in each treatment, further emphasizing the diversity and complexity of root exudate metabolism in response to Cd stress. Subsequently, a heatmap was generated to better investigate the relative changes of root exudates after Cd stress at different concentrations (Figure 3). It was observed that most alkaloids, vitamin B substances, flavonoids, terpenoids, phenolics, lipids, and organic acids were significantly upregulated, while some amino acids showed a downregulation in the Low group. In contrast, only a few root exudates exhibited significant changes in the High group.

MetaboAnalyst (version 6.0) was employed to investigate the changes in metabolic pathways for the identified differential metabolites (Figure S3). The Low/CK and High/CK groups exhibited comparable patterns of tyrosine metabolism, purine metabolism, and isoquinoline alkaloid biosynthesis. In addition, β-alanine metabolism, phenylpropanoid biosynthesis, and phenylalanine metabolism were the key pathways that were enriched in the Low/CK group. The fact that the Low/CK group exhibited more metabolic pathways than the High/CK group suggests that Cd stress had a considerable impact on the metabolic processes of rice root exudates, causing damage to the root system.

### 2.3. Effect of Cd Stress on Rhizosphere Soil Microbial Communities

The Chao1 index is recognized for its effectiveness in estimating species richness, with higher values indicating greater species diversity. The Shannon index was applied to measure species diversity, offering insights into the evenness and distribution of different species across the samples. This study presented a comparative analysis of microbial abundance and diversity in the rhizosphere soil between the blank soil control group and the soils contaminated with different Cd concentrations (Figure S4A,B). Compared with the Blank group, the High group showed a significant increase in microbial abundance (*p* < 0.05). Moreover, compared to the Low group, the High group exhibited a significant increase in abundance and diversity (*p* < 0.05). Collectively, these findings indicate that both rice cultivation and cadmium stress have the potential to significantly impact the abundance and diversity of soil microbial communities within the rhizosphere.

To evaluate the similarities and differences of the soil bacterial community composition under different treatments, principal coordinate analysis (PCoA) was used based on the Bray–Curtis algorithm (Figure S4C). The treatment groups, categorized by Cd application and concentration, were arranged along the first principal component from left to right. Distinct separations between these groups were observed, suggesting significant differences in their microbial compositions. Venn diagrams were employed to evaluate the particular makeup, overlap, and similarity among soil microbial communities under different treatment conditions (Figure S4D). The Blank, CK, Low, and High groups uniquely hosted 3099, 3892, 3538, and 4579 amplicon sequence variants (ASVs), respectively. Notably, the High group showed the largest number of unique ASVs (4579), suggesting Cd stress promoted niche differentiation, potentially enriching metal-resistant taxa. The minimal shared ASVs between Blank/Low (264) and Blank/High (215) vs. Blank/CK (504) imply Cd exposure drastically shifted communities away from both the unplanted (Blank) and uncontaminated (CK) states. The Blank, CK, Low, and High groups shared 1285 common ASVs, which likely represent the “core microbiome” essential for basic soil functioning and whose persistence under Cd stress suggests metabolic plasticity.

**Figure 4:**
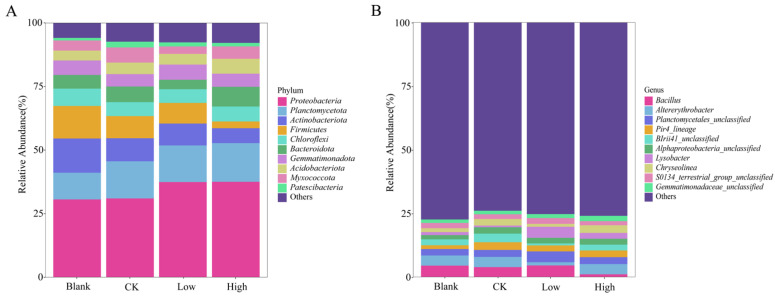
The modulation of soil bacterial communities by different concentrations of Cd stress. (**A**) Relative abundance of the major phyla of bacterial communities. (**B**) Relative abundance of the major genera of bacterial communities. The treatment groups include the blank group, the control (0 mg kg^−1^), low Cd concentration (2 mg kg^−1^), and high Cd concentration (10 mg kg^−1^).

The taxonomic composition of soil bacterial communities in different treatment groups was further evaluated (Figure 4A,B). Proteobacteria (30.62~37.5%) was the most dominant phylum in the four treatment groups. Notably, while *Proteobacteria* showed similar relative abundances between the Low (37.45%) and High (37.50%) groups, and between the Blank (30.62%) and CK (31.00%) groups, their overall abundance increased under Cd stress (Low/High vs. Blank/CK), suggesting significant Cd tolerance in this phylum. With the increasing concentration of Cd, a significant reduction was noted in the relative amount of Actinobacteriota (5.86~13.43%) and Firmicutes (2.64~12.80%). When the bacterial composition of each treatment group was analyzed at the genus level, *Bacillus* was overwhelmingly dominant in the soils of the three treatments (Blank—4.49%, CK—3.92%, Low—4.59%), while it was significantly reduced to 1.08% in the High group. However, the *Pir4_lineage* was more abundant in the CK, Low, and High groups (3.02%, 2.37%, and 2.64%) than in the Blank group (1.49%). Differences in some small proportions in the bacterial groups *Altererythrobacter* (1.03~4.02%), *Lysobacter* (0.54~4.35%), *Chryseolinea* (0.77~3.00%), and *Pirellula* (0.91~2.22%) were also observed between the four treatment groups of rice.

**Figure 5:**
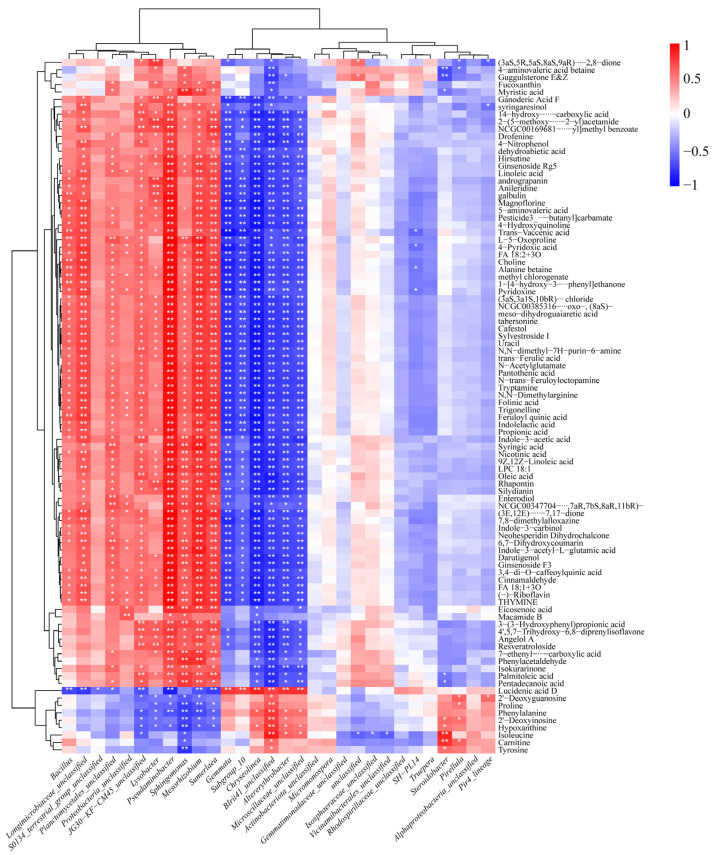
The clustered correlation heatmap between genus-level bacterial communities and differential root exudates. Note: significant correlation between bacteria and differential metabolites: * *p* < 0.05, ** *p* < 0.01.

The relationship between the genus-level bacterial communities and the root exudates was analyzed by means of correlation analysis based on the Pearson correlation coefficients (Figure 5). The genera *Pseudaminobacter*, *Hydrogenophaga*, and *Sumerlaea* were significantly positively correlated (*p* < 0.01) with most root exudates, including pyridoxine, trigonelline, and choline. This observation suggested that these bacterial communities, originating from root exudates, might influence the host plant. To elucidate the interactions among different microbial communities, this study calculated the abundance of the top 30 microbial genera at the genus level and subsequently analyzed the correlation and significant *p*-values between pairwise dominant microbial communities (Figure S5). The analysis demonstrated a robust association between *Pseudaminobacter* and *Hydrogenophaga*, suggesting a potential mechanism of mutual recruitment between these two genera.

**Figure 6:**
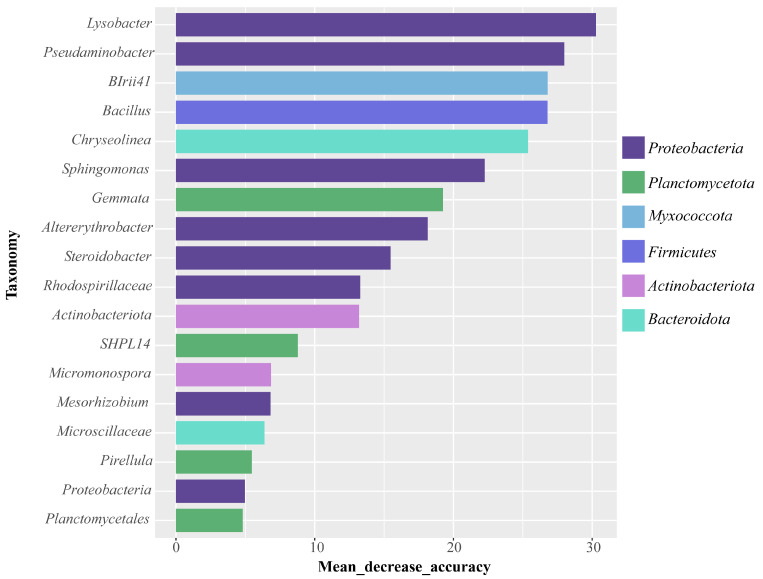
Bacterial biomarkers predicted by the random forest model. Different colors denote the phylum affiliation of each order.

Random forest, an efficient machine-learning technique, could be employed for the screening of biomarkers based on the contribution of mean accuracy. In this study, the random forest model was employed for screening biomarkers at the genus level, and a total of 18 biomarkers were identified (Figure 6). Among the identified biomarkers, a total of 6 bacterial genera were selected as the most predictive biomarkers based on a decrease in mean accuracy of >4%, which included *Lysobacter*, *Pseudaminobacter*, *Sphingomonas*, *Chryseolinea*, *BIrii41*, and *Bacillus*. These bacterial genera represented four phyla: Proteobacteria (30.62~37.5%), Bacteroidota (3.73~7.76%), Myxococcota (2.94~5.96%), and Firmicutes (2.64~12.80%). Among them, *Lysobacter*, *Pseudaminobacter*, and *Sphingomonas* were affiliated with Proteobacteria, which have been identified as the key biomarkers for monitoring cadmium contamination due to their relative abundance and predictive significance.

In order to explore the functional composition information of differential species in soil microbial communities of the different treatment groups, PICRUSt2 functional prediction analysis [30] and Statistical Analysis of Metagenomic Profiles (STAMP) [31] were utilized for visualization. Functional annotation was performed using the Clusters of Orthologous Groups (COG) database. As illustrated in Figure S6, the results of the *t*-test (*p* < 0.05) revealed significant differences in the functional configuration of microbial communities between the experimental groups and the CK group. In the Low group, only the number of proteins associated with the spore coat protein U (SCPU) domain was found to be significantly lower in contrast to the CK group, while other protein functions were higher than those in the CK group. In the High group, the phosphotransferase system was considerably lower relative to the CK group, indicating that the absorption of carbohydrates was significantly affected by the bacteria present in the soil of the High group. In contrast, the peptidoglycan/xylan/chitin deacetylase and the predicted unusual protein kinase regulating ubiquinone biosynthesis exhibited significantly elevated levels in the CK group. The former was capable of modulating carbon–hydrogen bonds in multiple compounds, while the latter catalyzed the phosphorylation of amino acid residues in proteins. The results of functional prediction showed that the rhizosphere microbial communities developed different metabolic strategies in response to different concentrations of Cd stress, and the microbial communities’ function changed to improve soil carbon utilization and boost soil microbial metabolic activity.

## 3. Discussion

### 3.1. Changes in Rice Root Exudate Patterns: Impacts on Cd Uptake and Translocation as Well as Self-Repair

Heavy metals could be enriched in plants and transported to aboveground tissues through the root system [32]. Previous studies demonstrated that the root system serves as a barrier to prevent the translocation of Cd to aboveground parts, thereby safeguarding the stem, leaves, and fruits from toxic effects [33]. In our previous research, the Cd content in rice tissues of different treatment groups was examined [34], indicating that under the influence of migration behavior, Cd was enriched and accumulated to varying degrees in different tissues.

It is worth noting that the Cd content in root tissues was significantly higher than that in aboveground parts, and leaf tissues exhibited the lowest Cd content. This indicates that the migration pattern of Cd in rice was from bottom to top, with root tissues exposed to Cd-contaminated areas showing higher accumulation. It was reported that approximately 70–80% of Cd was retained in the root system, while a minor fraction moved to the aboveground parts [7]. Similarly, our study identified that in the treatment groups, the PTI was significantly lower than the STI, which might have been attributed to a portion of root exudates chelated with Cd, leading to its substantial accumulation in roots and hindering Cd translocation.

In this study, it was found that the metabolic profiles of rice root exudates underwent significant changes across different Cd concentrations. These changes were predominantly observed in the secretion patterns of diverse compounds, encompassing amino acids, organic acids, phenolic compounds, and alkaloids. One of the most significant patterns observed in this study was the decreased release of tyrosine and proline when rice was under both high and low Cd stress, which might have been part of the response of rice to abiotic stress. These amino acids possessed potential chelating sites capable of binding heavy metal ions [35] and could have engaged in the formation of cyclic chelates with Cd by two O atoms from the α-COO- and side chain-COO- groups [36], thereby diminishing the absorption of cadmium by the rice root system from the surrounding environment, along with the transportation of cadmium via roots to other parts of rice. Therefore, with the increase in cadmium content, the amino acids in the root exudates of rice were gradually consumed, providing a relatively reasonable explanation for the significant increase in Cd accumulation and the decrease in the PTI in rice roots under Cd stress to some extent. It was interesting that organic acids (ferulic acid and syringic acid) exhibited a secretion pattern similar to amino acids when coping with Cd stress, suggesting that organic acids in root exudates also possess the function of alleviating Cd toxicity [6]. Additionally, the variation in root exudates also represented the adjustment of rice’s ability to self-repair as a reaction to environmental pressures. It is well-established that the enrichment of heavy metals, particularly Cd, results in the induction of oxidative stress in rice, compelling the plant to modulate its metabolic activities to accomplish self-repair. Consequently, the content of primary antioxidant constituents, such as phenolic (4-nitrophenol and syringaresinol) and alkaloid (alanyl betaine and magnoflorine), in rice root exudates decreased with the addition of Cd, thereby neutralizing the reactive oxygen species (ROS) induced by Cd stress [37].

### 3.2. Effects of Cd Stress on the Rhizosphere Microbial Communities

In this study, the high-throughput 16S rRNA sequencing technology was employed to elucidate the changes in microbial assemblages in the rhizosphere coping with Cd stress. This technique enabled the precise observation of slight alterations in microbial community composition, providing novel biological indicators for the assessment of soil health and Cd contamination. The results of this experiment demonstrated that the abundance and diversity of microbial communities in the rhizosphere soil were significantly changed after planting rice and Cd contamination.

Microbial assemblages characterized at the phylum level were composed of microflora with similar functional, morphological, and metabolic characteristics. In this experiment, Proteobacteria, Planctomycetota, and Actinobacteriota were the preponderant bacterial phyla. Among them, the abundances of Proteobacteria and Actinobacteriota fluctuated significantly in the different treatment groups. In soils under Cd stress, the microbial communities that undergo significant changes are typically categorized as Cd-resistant and Cd-sensitive types [38]. For instance, the phylum Proteobacteria has been frequently reported to maintain its dominance under a Cd-enriched environment, perhaps owing to the multitude of Cd-resistant varieties within the phylum [39]. The proportion of Proteobacteria in the Low and High groups showed a marked increase compared to the Blank and CK groups, which might have been attributed to their acclimatization and resilience to Cd-rich environments [40]. Additionally, the Proteobacteria were capable of producing antioxidant enzymes that aided in the plant’s resistance to oxidative stress and enhanced its tolerance to heavy metals, as substantiated by prior research [41]. As Actinobacteriota are very sensitive to heavy metal pollution, the abundance of Actinobacteriota serves as a crucial indicator in assessing whether the composition of soil microbial communities is conducive to plant growth. The relative abundance of Actinobacteriota decreased gradually as the degree of Cd stress intensified. This observation, in conjunction with our previous study [34], suggested that high Cd concentration inhibited the growth and activity of Actinobacteriota, potentially weakening the resistance and reducing the biomass of the root system.

Using random forest prediction, six bacterial genera were identified as the most representative biomarkers across the different treatment groups. Notably, their abundances peaked in the low-concentration group. The majority of these markers (*Bacillus*, *Lysobacter*, *Pseudaminobacter*, and *Sphingomonas*) have been reported to possess Cd resistance, which was consistent with their enrichment trend in the low-concentration group. The enrichment of metal-resistant genera (e.g., *Lysobacter*, *Sphingomonas*) in the Cd-treated groups may imply co-selection of antimicrobial traits. For instance, *Lysobacter* is known to produce lytic enzymes and antibiotics that suppress competing microbes [42], providing an ecological advantage under combined Cd and biotic stresses. This dual functionality (metal resistance and antimicrobial activity) could further enhance their dominance in the rhizosphere under low Cd exposure (Low group). Unfortunately, their abundances were reduced in the group with high concentrations, possibly due to the excessive Cd levels exceeding their tolerance thresholds. The study by Ma et al. [43] indicated that under high-concentration heavy metal stress, the abundance of certain microbial communities decreased, possibly because it exceeded their tolerance thresholds, which was consistent with our conclusions. Secondly, under low-concentration Cd stress, plants released a substantial amount of root exudates serving as carbon and nitrogen sources to recruit Cd-resistant bacteria, while this process was inhibited under high-concentration Cd stress, leading to intense competition among different bacterial genera in the limited carbon and nitrogen resource environment, further contributing to the decrease in their abundance. Interestingly, the relative abundance of the *Pir4_lineage* in the Blank group was 1.49%, whereas the abundance increased significantly in the CK, Low, and High groups, with 3.02%, 2.37%, and 2.64%, respectively. The increase in the *Pir4_lineage* after planting rice might have been due to its recruitment by rice to promote its growth.

### 3.3. Ecological Regulatory Mechanisms of Microbial Communities Recruited by Rice Root Exudates Under Cd Stress

This study not only analyzed the changes in rice root exudates and rhizosphere microbial communities, but also, more importantly, it explored the potential ecological regulatory mechanisms that might exist when it is exposed to Cd. In this study, stress from Cd significantly altered the composition of rice root exudates. The alteration of root exudates was not only reflected in the protection of the plant itself, but also had an important effect on the inter-root microbial community. During heavy metal stress, rice and soil microbial communities formed a cooperative defense against the adverse effects of environmental changes and coregulated the plant–root–microbe system [44,45]. Griffiths et al. [46] found that the addition of amino acids to soil had the capacity to alter the structure of soil microbial communities. Other root secretions and secondary metabolites also showed a positive response. Studies have indicated that phenolic compounds act as substrates of soil bacteria and stimulate the growth of certain microorganisms, thereby altering the makeup of the soil microbial ecosystem [47]. Integrating the above perspective of plants releasing root exudates in response to Cd stress, we proposed that the emission of root exudates is facilitated by plants, serving as a signal to recruit probiotic microorganisms. Soil microorganisms migrate toward the roots by recognizing the signal and form a new rhizosphere defense system in conjunction with the pre-existing microbial communities.

Through the random forest model analysis, Proteobacteria (*Lysobacter*, *Pseudaminobacter*, and *Sphingomonas*) were established as bioindicators reflecting the degree of plant response to Cd stress, which not only represents the degree of response to Cd pollution, but is also involved in the tolerance and detoxification processes of plants [48]. We established that there was a notably strong positive link between *Pseudaminobacter* and vitamins, organic acids, and flavonoids, which had been established to function as signaling molecules or nutritional sources, thereby recruiting beneficial microbes from the soil. Previous research had found that *Pseudaminobacter* was sensitively responsive to peanut root exudates [49]. Furthermore, studies have indicated that the introduction of *Pseudaminobacter* to peanuts could boost the release of root exudates [50]. Consequently, we suggested that under Cd stress, the plants could recruit beneficial bacteria to combat Cd stress by releasing secondary metabolites as signaling molecules, and these beneficial bacteria, in turn, influence the exudation of root secretions. This process had the potential to capture Cd in the roots and prevent its upward translocation. Similarly, *Sphingomonas* and *Lysobacter* showed a marked positive association with the majority of root exudates, and it was demonstrated that *Sphingomonas* and *Lysobacter* assist plants in coping with Cd stress through various mechanisms [51]. In summary, our research indicated that root exudates possess the capacity to recruit *Pseudaminobacter*, *Sphingomonas*, and *Lysobacter*, thereby improving rice tolerance in the Cd environment. Furthermore, it was revealed that the levels of beneficial bacteria and root exudates, which contribute to resistance against Cd stress, were significantly upregulated in the Low group in comparison to the control group. This could further lead to increased stress effects in rice in the High group, but the specific mechanisms involved remain to be determined. In summary, this study not only enhanced our comprehension of the ecological adaptability mechanisms of rice in the face of heavy metal stress, but also offered novel strategies and tools for agricultural ecological regulation and soil health management. Future research should explore the practical applications and examine how they might assist in developing more efficacious methods for plant protection and management.

## 4. Materials and Methods

### 4.1. Experimental Design

The present experiment was conducted within the greenhouse facilities of Guilin University of Technology (N25°03′05″, E110°170′50″). Rice seeds (TanLiangYou215, TLY215, purchased from Hunan Tannong Huayuan Seed Industry Co., Ltd., Xiangtan, China) with uniform weight, consistent size, and no surface damage were selected, and these seeds were surface-sterilized with 2% *v*/*v* NaClO (effective chlorine ≥ 8.0%, AR, purchased from Guangzhou Xilong Chemical Co., Ltd., Guangzhou, China) after stirring on a magnetic stirrer at 200 rpm for 10–15 min, followed by thorough rinsing with deionized water three to five times to remove any residual NaClO. The seeds were placed in a dark environment at 30 °C in a light incubator. After two days of incubation, only those seeds that had successfully ruptured their seed coats and demonstrated robust germination were selected for subsequent stress experiments.

The planting pots (50 cm length × 20 cm width × 14 cm height) were filled with 6 kg of a soil mixture composed of equal parts nutrient soil (loamy texture), organic matter soil (peat-based, natural organic matter ≥30%), and sand and gravel (particle size, 0.5–2 mm, quartz-dominant), each by weight, in a 1:1:1 ratio, and subsequently treated with CdCl_2_ (99.9%, AR, purchased from Guangzhou Xilong Chemical Co., Ltd.). Three treatment groups with different Cd concentration levels were established. The added concentrations were 0 mg·kg⁻^1^ (CK, the control group without additional Cd treatment), 2 mg·kg⁻^1^ (Low), and 10 mg·kg⁻^1^ (High). Furthermore, a blank group with untreated soil was set up, which consisted of the same soil mixture (nutrient soil, organic matter soil, and sand and gravel in a 1:1:1 ratio) but without the addition of CdCl_2_, and no rice was planted. The Blank group was included in microbial community analysis to characterize the native soil microbiome as a baseline control, while it was excluded from root exudate analysis since no rice plants were present to produce root exudates. Each group was sprayed daily with 300 mL of deionized water. After 10 days, sixty-five rice seeds were selected based on uniform size, weight, germination vigor, physical integrity, and absence of damage, ensuring high performance and consistency across all treatment groups. The selected seeds were then planted, and the soil was covered with vermiculite. During germination, at the early stage, rice was cultured only with a limited amount of deionized water. During the period from the one-leaf to the three-leaf stage, a 25% nutrient solution (prepared from Yoshida rice nutrient dry powder, purchased from Coolaber, Beijing, China) was applied. Once the plants reached the three-leaf stage, they were then grown using a 50% nutrient solution. Throughout the cultivation period, the water level was consistently kept at a depth of 2–3 cm. Each treatment group was replicated twice. After a 90-day Cd treatment, the whole plants with their roots were carefully extracted from the soil and then gently shaken to remove loosely adhering soil (Figure S1). The rhizosphere soil and plant-free blank soil were collected. These samples were then sifted through a 20-mesh sieve, flash-frozen in liquid nitrogen, and stored at −80 °C. Each treatment group included six biological replicates. The Cd concentrations in rice tissues were determined by ICP-MS following the digestion and measurement procedures described in our previous study [34].

### 4.2. Analysis of Root Exudates

After 90 days of Cd stress, the roots of each collected plant were meticulously rinsed multiple times with deionized water to eliminate any residual soil particles. For each treatment group, five plants with similar growth potential were selected and transferred into a 100 mL centrifuge tube, which was filled with 40 mL sterile ultrapure water and wrapped in tin foil away from light. After 48 h, the solution was filtered through a 0.22 μm mixed cellulose membrane, and the resulting samples were stored at −20 °C. Quality control (QC) samples were generated by amalgamating 20 μL of each extracted sample, with six biological replicates conducted for each experimental group.

The metabolic profile was analyzed in ESI positive/negative ion modes using a Shimadzu LC-20A (Shimadzu, Shanghai, China) equipped with a Sciex QTOF/MS (TripleTOF 5600+, SCIEX, Framingham, MA, USA). An XSelect^®^ HSS T3 column (150 mm × 2.1 mm × 3.5 μm, Waters, Milford, MA, USA) was employed to separate the metabolites in the extract. The mass spectrometer was set to scan from 50 to 1000 *m*/*z*. The mobile phases comprised formic acid (0.1%, *v*/*v*) and 5 mM ammonium formate and were used in the positive and negative ion modes, respectively, while pure acetonitrile served as the mobile phase B in both modes. The gradient elution profile was as follows: from 0.00 to 3.00 min, 1% B; from 3.01 to 24.00 min, a gradient from 1% to 100% B; from 24.01 to 32.00 min, 100% B; and from 32.01 to 37.00 min, returning to 1% B. The flow rate was 0.3 mL min^–1^, with an injection volume of 2 μL. For positive ion detection, the source voltage was maintained at 5500 V and the collision energy at 30 V. Conversely, for negative ion detection, the source voltage was −4500 V and the collision energy was −30 V.

The raw data were converted into the ABF format utilizing AnalysisBaseFileConverter (version 2019) and subsequently analyzed using MS-DIAL software (version 4.36). This analytical process encompassed noise filtering, peak identification, overlapping peak analysis, peak alignment, and peak filling. The preprocessed data, which comprised retention time, mass-to-charge ratio, and peak area, were compiled into a matrix. Following this, metabolites were identified through the use of public databases such as MassBank, LipidBlast, and MetaboBase, alongside chemical standard databases. The identification procedure was based on retention time, mass accuracy, and MS/MS fragmentation spectra, employing a cutoff score of 80% and mass tolerances of 0.05 Da for MS1 and 0.10 Da for MS2, respectively. The raw data from both positive and negative ion modes were processed according to the aforementioned methodology and combined for subsequent statistical analysis.

### 4.3. DNA Extraction and Bacterial Sequencing

The total microbiome DNA from the rhizosphere soil samples of rice was extracted utilizing the CTAB method. The DNA extraction procedure was as follows. Initially, rhizospheric soil samples were carefully collected following specific sampling protocols. Subsequently, 1 mL of pre-heated (65 °C) CTAB extraction buffer was added to the centrifuge tube containing the sample. The sample was thoroughly vortex-mixed until it was homogeneously suspended, and then incubated in a 65 °C water bath for 60 min. During the water bath incubation, the sample was gently shaken 2–3 times to ensure complete lysis. After incubation, the sample was allowed to cool to room temperature. Next, the sample was centrifuged at 8000 rpm for 5 min, and the resulting supernatant was carefully transferred to a new sterile centrifuge tube. Then, 800 μL of chloroform–isoamyl alcohol (24:1) was added to the tube, which was then gently inverted 100 times (taking care to avoid mechanical shearing of the DNA). The mixture was centrifuged at 12,000 rpm for 20 min. The supernatant was carefully transferred to another new tube, and the chloroform–isoamyl alcohol extraction step was repeated. Thereafter, 400 μL of the supernatant was transferred to a sterile centrifuge tube. Subsequently, 2/3 of the supernatant volume of isopropanol and 1/10 of the supernatant volume of 3M sodium acetate were added. The components were thoroughly mixed, and the tube was placed at −20 °C for 1 h. The sample was then centrifuged at 12,000 rpm for 10 min. After centrifugation, the supernatant was discarded, and any remaining liquid was removed by brief centrifugation and careful aspiration using a yellow pipette tip. The DNA pellet was air-dried at room temperature for 10 min until it attained a semi-transparent appearance. Finally, 50–100 μL of sterile ddH_2_O containing 10 mg·mL^−1^ RNase was added to dissolve the DNA pellet. The sample was incubated in a 37 °C water bath for 1 h for RNase digestion and then stored at −20 °C for long-term preservation.

The extraction’s success was confirmed by agarose gel electrophoresis, in anticipation of the following PCR amplification steps. The V3–V4 regions of bacterial 16S rRNA genes were amplified using primers 341F (5′-CCTACGGGGNGGCWGCAG-3′) and 805R (5′-GACTACHVGGGTATCTAATCC-3′) to enable microbial community profiling through Illumina sequencing [52]. PCR amplification was conducted in a total reaction volume of 25 μL, comprising 25 ng of template DNA, 12.5 μL of PCR Premix, 2.5 μL of each primer, and PCR-grade water to achieve the desired volume. The thermocycling protocol included an initial denaturation step at 98 °C for 30 s, followed by 32 cycles consisting of 10 s of denaturation at 98 °C, 30 s of annealing at 54 °C, and 45 s of extension at 72 °C. This was concluded with a final elongation step at 72 °C for 10 min. The resulting PCR products were verified using 2% agarose gel electrophoresis. Throughout the DNA extraction process, ultrapure water was employed as a means of eliminating the potential for false positives. The PCR products underwent purification utilizing AMPure XT beads (Beckman Coulter Genomics, Danvers, MA, USA) and were quantified by Qubit (Invitrogen, Carlsbad, CA, USA). The size and quantity of the amplicon library were evaluated with an Agilent 2100 Bioanalyzer (Agilent, Santa Clara, CA, USA) and the Library Quantification Kit for Illumina (Kapa Biosciences, Woburn, MA, USA), respectively. Ultimately, the libraries were sequenced on a NovaSeq PE250 platform (Illumina, CA, USA).

Paired-end reads were allocated to samples according to their distinct barcodes and subsequently trimmed to remove the barcode and primer sequences. The cleaned reads were merged using the FLASH software (version 1.2.8) [53]. To ensure the quality of the raw data, Fqtrim (version 0.94) was employed to generate paired-end clean tags. The data were processed to eliminate chimeric sequences with Vsearch (version 2.3.4) [54]. Then, DADA2 (version 2019.7) was applied for dereplication to yield feature tables and feature sequences; α-diversity and β-diversity indices were derived by normalizing the data to an equal number of sequences for random sampling. The abundance of features was normalized on the basis of their relative abundance in each sample, classified in accordance with the SILVA database (release 138). The R package (version 3.5.2) was utilized for the statistical analysis of common and endemic species, as well as for evaluating the community composition.

### 4.4. Statistical Analysis

Unsupervised principal component analysis (PCA) was employed to provide a comprehensive overview of the metabolic variation and elucidate the similarities and differences among rice samples with varying concentrations of Cd. Differential metabolites were identified utilizing partial least squares discriminant analysis (PLS-DA) and *t*-tests, with VIP > 1, *p* < 0.05 as selection thresholds. The significance of differences was assessed through one-way analysis of variance (ANOVA) using SPSS version 26.0, with a significance level established at *p* < 0.05. Heatmap visualization and the volcano plot were completed using the web platform https://www.omicstudio.cn/tool (accessed on 5 February 2024). Furthermore, the metabolic pathway alterations of the identified characteristic differential metabolites were analyzed using the MetaboAnalyst platform (https://www.metaboanalyst.ca, accessed on 9 February 2024) and utilizing references from the rice database available in the Kyoto Encyclopedia of Genes and Genomes (KEGG).

The formulas for calculating the PTI and the STI are as follows:

(1) $$\mathrm{Primary}\;\mathrm{Transport}\;\mathrm{Index}=\frac{\mathrm{heavy}\;\mathrm{metal}\;\mathrm{content}\;\mathrm{in}\;\mathrm{stem}}{\mathrm{heavy}\;\mathrm{metal}\;\mathrm{content}\;\mathrm{in}\;\mathrm{root}\;\mathrm{system}}$$

(2) $$\mathrm{Sec}\mathrm{ondary}\;\mathrm{Transport}\;\mathrm{Index}=\frac{\mathrm{heavy}\;\mathrm{metal}\;\mathrm{content}\;\mathrm{in}\;\mathrm{leaves}}{\mathrm{heavy}\;\mathrm{metal}\;\mathrm{content}\;\mathrm{in}\;\mathrm{stem}}$$

## 5. Conclusions

Through the metabolomic technology and the high-throughput sequencing technology, this study comprehensively explored the impact of Cd stress on the composition of root exudates and rhizosphere microbial communities, as well as the interactions between them. The findings reveal that Cd stress had a marked impact on the composition of rice root exudates. These modified exudates selectively enriched beneficial bacterial taxa, such as *Lysobacter*, *Pseudaminobacter*, and *Sphingomonas* (all belonging to the phylum Proteobacteria), which exhibited a significant positive correlation with most root exudates, suggesting their pivotal roles in the plant defense system. Their presence reflected an adaptive response to Cd stress and indicates their potential involvement in plant tolerance and detoxification mechanisms. The findings offer novel insights into the ecological adaptation mechanisms of rice under heavy metal stress and provide potential biomarkers and microbial resources for agricultural environmental regulation.

## Data Availability

The original contributions presented in the study are included in the article; further inquiries can be directed to the corresponding authors.

## Supplementary Materials

The following supporting information can be downloaded at: https://www.mdpi.com/article/10.3390/plants14111695/s1, Table S1: Soil pH, Cd concentration, and growth height of rice (mean ± SD, n = 3). Table S2: Detailed information of differential metabolites among different groups. Figure S1: Representative images of rice plants under different cadmium (Cd) treatment conditions. From left to right: CK group, low-Cd treatment group, and high-Cd treatment group. Visible differences in growth and morphology between the groups are observed, reflecting the impact of Cd stress on rice development. Figure S2: Classification of 93 differential compounds in rice root exudates. Figure S3: Metabolic pathway analysis of root exudates with significant differences in (A) the low-treatment (Low) and (B) high-treatment (High) groups compared to the control (CK) group. Figure S4: (A) Chao1 index (index of mycorrhizal abundance); (B) Shannon index (index of mycorrhizal diversity); (C) principal coordinate analysis (PCoA); and (D) Venn’s analysis in rhizosphere soils. Blank: blank group; CK: control group; Low: 2 mg kg^−1^; High: 10 mg kg^−1^. Figure S5: Hierarchical clustering analysis of microbial communities at the genus level: correlation matrix of the top 30 most abundant genera. Figure S6: Statistical Analysis of Metagenomic Profiles (STAMP) (*p* < 0.05). (A) CK/Blank; (B) CK/Low; (C) CK/High. The significant difference function between the two groups was obtained by this analysis.
