# Supplementary material for: Effects of Cadmium Stress on Root Exudates and Soil Rhizosphere Microorganisms of Rice (Oryza sativa L.) and Its Ecological Regulatory Mechanisms
Source: Plants (Basel). 2025 Jun 1;14(11):1695. doi: 10.3390/plants14111695 (PMC12157188; doi:10.3390/plants14111695)
Supplement: Supplementary file 1 [file plants-14-01695-s001.zip › plants-3638156-supplementary.pdf]

Appendix. Supplementary Materials for

Effects of cadmium stress on root exudates and soil rhizosphere microorganisms of rice (*Oryza sativa* L.) and its ecological regulatory mechanisms.

Siqi Lin[a,†], Qing He[a,†], Mingxia Zhang[b], Yingyi Huang[a], Huahong Liu[a], Qi'er Mu[a], Sheng Wang[a,*], Jinfang Nie[a,*]

[a] College of Chemical and Bioengineering, Guilin University of Technology, Guilin 541004, China

[b] Guangxi Research Institute of Tea Science, Guilin 541004, China

To whom correspondence should be addressed:

* Corresponding author.

E-mail: Niejinfang@glut.edu.cn (J.F. Nie), wangsheng@glut.edu.cn (S. Wang)

Table S1

Soil pH, Cd concentration, and growth height of rice (Mean ± SD, n = 3).

		CK	Low	High
Soil pH		7.15 ± 0.03^{a}	6.95 ± 0.01^{b}	6.80 ± 0.01^{c}
Cd concentration in rice ($mg \cdot kg^{-1}$)	Roots	1.45 ± 0.27^{c}	15.00 ± 1.6^{b}	58.34 ± 2.67^{a}
	Stems	0.22 ± 0.03^{c}	1.87 ± 0.18^{b}	3.72 ± 0.38^{a}
	Leaves	0.10 ± 0.01^{c}	1.20 ± 0.2^{b}	2.97 ± 0.18^{a}
Above-ground height(cm)		71.1 ± 5.93^{b}	86.98 ± 8.29^{a}	66.96 ± 6.08^{b}

Table S2

The detailed information of differential metabolites among different groups.

NO.	Metabolite name	Average Rt(min)	Average Mz	Formula	Ontology	Reference m/z
1	Uracil	1.193	113.0331	$C_4H_4N_2O_2$	Pyrimidones	113.0346
2	Tyrosine	2.751	182.0796	$C_9H_{11}NO_3$	Tyrosine and derivatives	182.0812
3	Tryptamine	7.714	161.1064	$C_{10}H_{12}N_2$	Tryptamines and derivatives	161.1073
4	Trigonelline	1.137	138.0573	$C_7H_7NO_2$	Alkaloids and derivatives	138.055
5	Trans-Vaccenic acid	22.936	281.2487	$C_{18}H_{34}O_2$	Long-chain fatty acids	281.2486
6	trans-Ferulic acid	7.556	177.0534	$C_{10}H_{10}O_4$	Hydroxycinnamic acids	177.0546
7	THYMINE	3.362	127.0501	$C_5H_6N_2O_2$	Hydroxypyrimidines	127.0502
8	tabersonine	6.982	337.1859	$C_{21}H_{24}N_2O_2$	Plumeran-type alkaloids	337.19
9	Syringic acid	8.624	199.0572	$C_9H_{10}O_5$	Gallic acid and derivatives	199.06
10	syringaresinol	12.089	417.1446	$C_{22}H_{26}O_8$	Furanoid lignans	417.1555
11	Sylvestroside I	10.997	771.2349	$C_{33}H_{48}O_{19}$	Iridoid O-glycosides	771.26
12	Silydianin	9.771	483.124	$C_{25}H_{22}O_{10}$	Flavanones	483.13
13	Rhapontin	11.034	443.127	$C_{21}H_{24}O_9$	Stilbene glycosides	443.13
14	Resveratroloside	9.836	413.1184	$C_{20}H_{22}O_8$	Stilbene glycosides	413.12
15	Pyridoxine	2.253	170.0805	$C_8H_{11}NO_3$	Pyridoxines	170.0812

16	Propionic acid	0.768	73.02904	$C_3H_6O_2$	Carboxylic acids	73.0295
17	Proline	1.008	116.0712	$C_5H_9NO_2$	Proline and derivatives	116.0706
18	Phenylalanine	5.839	166.0865	$C_9H_{11}NO_2$	Phenylalanine and derivatives	166.0863
19	Phenylacetaldehyde	9.672	121.0666	C_8H_8O	Phenylacetaldehydes	121.0648
20	Iprovalicarb	13.463	343.1885	$C_{18}H_{28}N_2O_3$	Toluenes	343.199
21	Pentadecanoic acid	20.955	241.2127	$C_{15}H_{30}O_2$	Long-chain fatty acids	241.2173
22	Pantothenic acid	6.523	220.1164	$C_9H_{17}NO_5$	Secondary alcohols	220.118
23	Palmitoleic acid	20.399	253.2129	$C_{16}H_{30}O_2$	Long-chain fatty acids	253.2173
24	Oleic acid	23.174	281.2496	$C_{18}H_{34}O_2$	Long-chain fatty acids	281.2486
25	N-Feruloyloctopamine	6.921	330.1309	$C_{18}H_{19}NO_5$	Hydroxycinnamic acids and derivatives	330.13
26	Nicotinic acid	1.621	124.0398	$C_6H_5NO_2$	Pyridinecarboxylic acids	124.0393
27	Neohesperidin Dihydrochalcone	9.205	635.1994	$C_{28}H_{36}O_{15}$	Flavonoid O-glycosides	635.19
28	Compound NP-000445	21.401	343.2246	$C_{20}H_{32}O_3$	Diterpenoids	343.224
29	NCGC00347704-02_C24H32O7_2H-Oxireno[1,10a]phenanthro[3,2-b]furan-10(11bH)-one, 5,7-bis(acetyloxy)-3,3a,4,5,6,7,7a,7b,8,8a-decahydro-4,4,7a,11-tetramethyl-, (1aS,3aR,5S,7S,7aR,7bS,8aR,11bR)-	16.931	415.2109	$C_{24}H_{32}O_7$	Naphthofurans	415.211
30	Compound NP-001068	10.553	473.1397	$C_{22}H_{26}O_{10}$	Iridoid O-glycosides	473.142
31	N-Acetylglutamate	2.332	190.068	$C_7H_{11}NO_5$	Glutamic acid and derivatives	190.071
32	N,N-Dimethylarginine	1.018	203.1489	$C_8H_{18}N_4O_2$	NA	203.1503
33	N,N-dimethyl-7H-purin-6-amine	5.756	164.093	$C_7H_9N_5$	6-alkylaminopurines	164.09
34	Myristic acid	19.533	227.2031	$C_{14}H_{28}O_2$	Long-chain fatty acids	227.2017
35	methyl chlorogenate	8.101	369.1169	$C_{17}H_{20}O_9$	/	369.1172
36	meso-dihydroguaiaretic acid	14.383	353.1705	$C_{20}H_{26}O_4$	Dibenzylbutane lignans	353.17

37	Magnoflorine	13.292	343.1834	$C_{20}H_{24}NO_4+$	Aporphines	343.17
38	Lucidenic acid D	17.656	515.2604	$C_{29}H_{38}O_8$	Triterpenoids	515.26
39	LPC 18:1	0.725	522.3675	$C_{26}H_{52}NO_7P$	Lipids	522.357
40	linoleic acid	10.72	303.2322	$C_{18}H_{32}O_2$	Lineolic acids and derivatives	303.23
41	L-5-Oxoproline	33.614	130.0636	$C_5H_7NO_3$	Alpha amino acids and derivatives	130.0499
42	Isoleucine	3.034	132.1016	$C_6H_{13}NO_2$	Isoleucine and derivatives	132.1019
43	Isokurarinone	16.933	439.201	$C_{26}H_{30}O_6$	8-prenylated flavanones	439.21
44	Indolelactic acid	6.745	204.0633	$C_{11}H_{11}NO_3$	Indolyl carboxylic acids and derivatives	204.0666
45	Auxistim G	6.041	303.0972	$C_{15}H_{16}N_2O_5$	Glutamic acid and derivatives	303.0986
46	Indole-3-acetic acid	6.733	174.0506	$C_{10}H_9NO_2$	Indole-3-acetic acid derivatives	174.0561
47	Hypoxanthine	5.608	137.0462	$C_5H_4N_4O$	Hypoxanthines	137.0458
48	Hirsutine	10.554	369.2282	$C_{22}H_{28}N_2O_3$	Corynanthean-type alkaloids	369.2173
49	Guggulsterone E&Z	19.406	313.2346	$C_{21}H_{28}O_2$	Androgens and derivatives	313.22
50	Ginsenoside Rg5	24.057	765.484	$C_{42}H_{70}O_{12}$	Triterpene saponins	765.4794
51	Ginsenoside F3	23.915	815.4919	$C_{41}H_{70}O_{13}$	Triterpenoids	815.4799
52	Ganoderic Acid F	23.898	593.2702	$C_{32}H_{42}O_9$	Triterpenoids	593.27
53	galbulin	1.025	379.1783	$C_{22}H_{28}O_4$	Aryltetralin lignans	379.19
54	Fucoxanthin	23.214	681.4127	$C_{42}H_{58}O_6$	Xanthophylls	681.41
55	Folinic acid	7.101	474.1684	$C_{20}H_{23}N_7O_7$	Tetrahydrofolic acids	474.1732
56	Feruloyl quinic acid	1.016	367.0962	$C_{17}H_{20}O_9$	Quinic acid and derivatives	367.102
57	FA 18:2+3O	12.823	327.2166	$C_{18}H_{32}O_5$	Oxidized fatty acids	327.2165
58	FA 18:1+3O	8.817	329.231	$C_{18}H_{34}O_5$	Oxidized fatty acids	329.2312
59	Enterodiol	21.296	301.1571	$C_{18}H_{22}O_4$	Dibenzylbutanediol lignans	301.1445
60	Eicosenoic acid	25.078	309.2762	$C_{20}H_{38}O_2$	Long-chain fatty acids	309.2799
61	Drofenine	17.382	318.2456	$C_{20}H_{31}NO_2$	Benzene and substituted derivatives	318.2428
62	dehydroabietic acid	19.058	299.2036	$C_{20}H_{28}O_2$	Diterpenoids	299.2016

63	Darutigenol	17.618	345.2366	$C_{20}H_{34}O_3$	Diterpenoids	345.24
64	Cinnamaldehyde	12.274	133.0631	C_9H_8O	Cinnamaldehydes	133.0648
65	Choline	10.858	105.1226	$[C_5H_{14}NO]^+$	Cholines	105.12
66	Carnitine	0.944	162.1115	$C_7H_{15}NO_3$	Carnitines	162.1125
67	Cafestol	18.326	339.1874	$C_{20}H_{28}O_3$	Naphthofurans	339.19
68	Anileridine	14.904	353.2319	$C_{22}H_{28}N_2O_2$	Phenylpiperidines	353.2224
69	Angelol A	9.84	399.1421	$C_{20}H_{24}O_7$	Coumarins and derivatives	399.14
70	andrograpanin	15.772	341.2044	$C_{20}H_{30}O_3$	Diterpene lactones	341.21
71	Alanine betaine	1.022	132.1139	$C_6H_{13}NO_2$	Alanine and derivatives	132.1019
72	Acanthoic acid	21.232	301.2173	$C_{20}H_{30}O_2$	Diterpenoids	301.2173
73	7,8-dimethylalloxazine	10.594	243.0839	$C_{12}H_{10}N_4O_2$	/	243.0877
74	6,7-Dihydroxycoumarin	0.832	177.0066	$C_9H_6O_4$	6,7-dihydroxycoumarins	177.0193
75	5-aminovaleric acid	1.025	118.0873	$C_5H_{11}NO_2$	Delta amino acids and derivatives	118.0863
76	4-Pyridoxic acid	6.271	182.0455	$C_8H_9NO_4$	Pyridinecarboxylic acids	182.0459
77	4-Nitrophenol	10.497	138.02	$C_6H_5NO_3$	Nitrophenols	138.0197
78	4-Hydroxyquinoline	10.49	144.0468	C_9H_7NO	Hydroquinolones	144.0455
79	4-aminovaleric acid betaine	1.029	160.1316	$C_8H_{17}NO_2$	/	160.1332
80	6,8-diprenylgenistein	10.914	429.1516	$C_{25}H_{26}O_5$	6-prenylated isoflavanones	429.17
81	3,4-di-O-caffeoylquinic acid	7.632	515.1193	$C_{25}H_{24}O_{12}$	/	515.1192
82	3-(3-Hydroxyphenyl)propionic acid	5.879	165.0574	$C_9H_{10}O_3$	Phenylpropanoic acids	165.0557
83	2'-Deoxyinosine	5.606	253.0932	$C_{10}H_{12}N_4O_4$	Purine 2'-deoxyribonucleosides	253.0931
84	2'-Deoxyguanosine	5.501	268.1014	$C_{10}H_{13}N_5O_4$	Purine 2'-deoxyribonucleosides	268.104
85	2-(5-methoxy-4,8,8-trimethyl-2-oxo-2,8,9,10-tetrahydropyrano[2,3-f]chromen-3-yl)-N-(pyridin-2-yl)acetamide	10.602	431.166	$C_{23}H_{24}N_2O_5$	Angular pyranocoumarins	431.16
86	CHEBI:65778	12.138	335.2263	$C_{20}H_{32}O_4$	Kaurane diterpenoids	335.2228

87	1-[4-hydroxy-3-(3-methylbut-2-enyl)phenyl]ethanone	14.508	205.1205	$C_{13}H_{16}O_2$	Alkyl-phenylketones	205.1223
88	(3E,12E)-3,12-dimethyl-8-methylidene-6,18-dioxatricyclo[14.2.1.0?,?]nonadeca-3,12,16(19)-triene-7,17-dione	20.202	351.1606	$C_{20}H_{24}O_4$	Diterpene lactones	351.1567
89	8-epi-Confertin	13.294	266.1734	$C_{15}H_{20}O_3$	Ambrosanolides and secoambrosanolides	266.1751
90	(3aS,3a1S,10bR)-3a-ethyl-5-(methoxycarbonyl)-13-methyl-1,2,3,3a,3a1,4,6,11,12,13-decahydroindolizino[8,1-cd]carbazol-13-ium chloride	13.533	354.2295	$C_{22}H_{29}N_2O_2+$	Aspidospermatan-type alkaloids	354.23
91	(-)-Riboflavin	8.489	377.142	$C_{17}H_{20}N_4O_6$	Flavins	377.1456

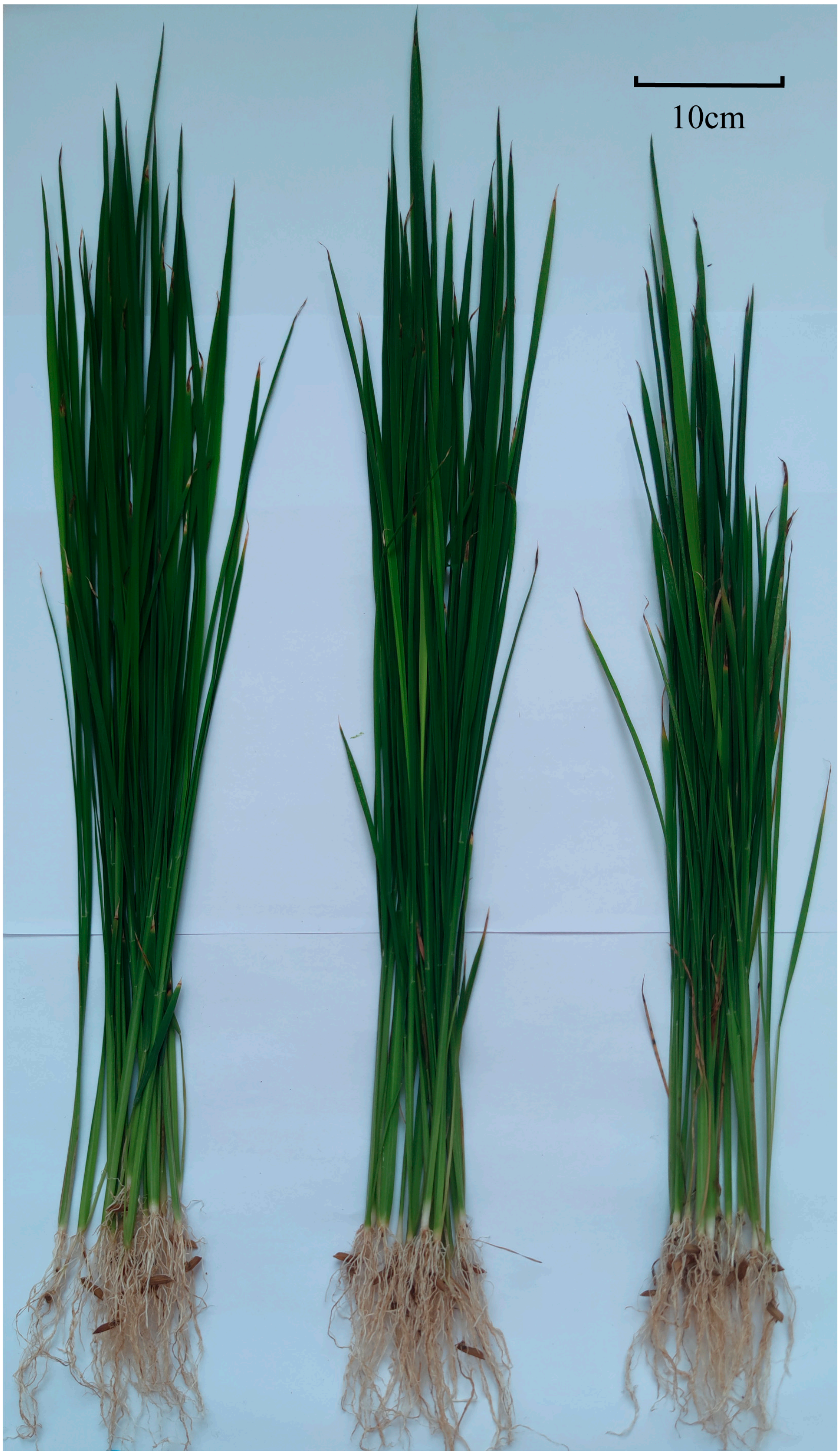

Figure S1. Representative images of rice plants under different cadmium (Cd) treatment conditions. From left to right: CK group, low Cd treatment group, and high Cd treatment group. Visible differences in growth and morphology among the groups are observed, reflecting the impact of Cd stress on rice development.

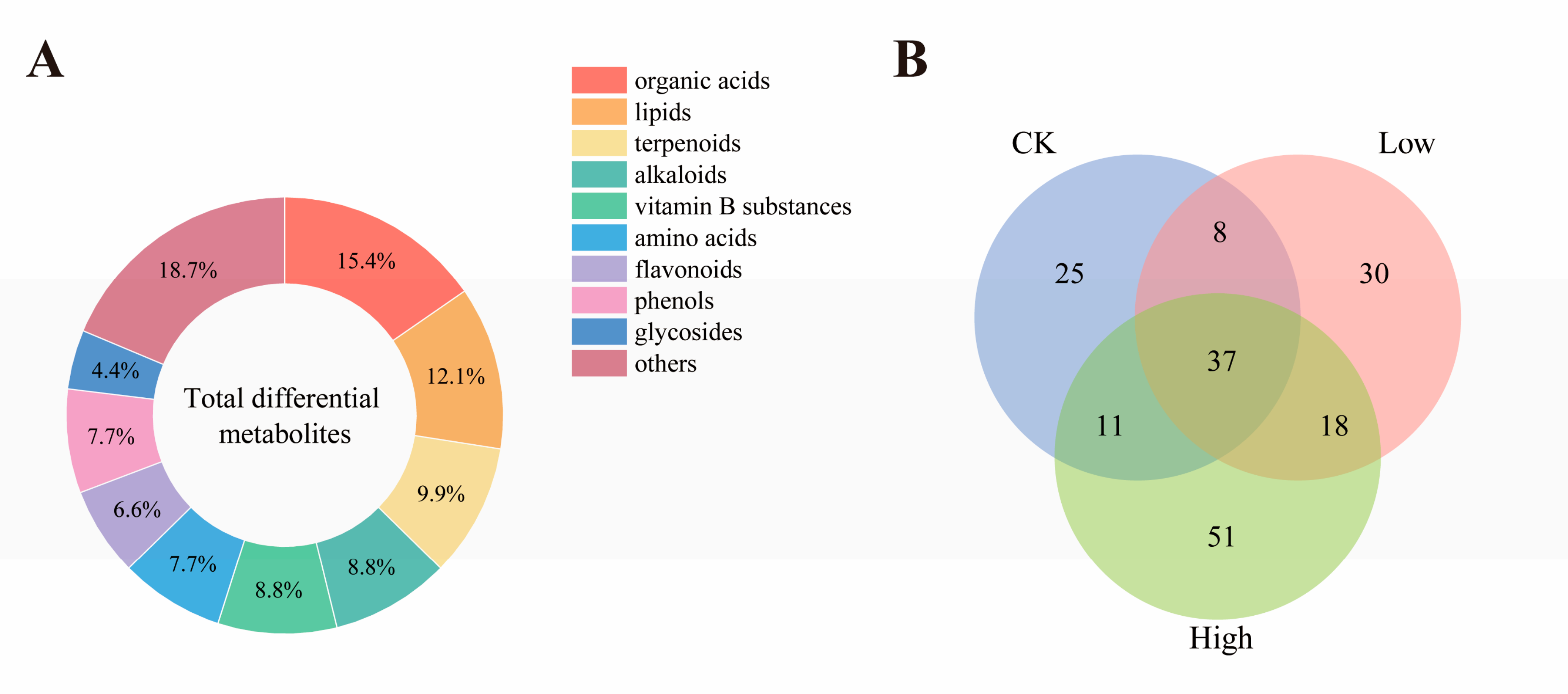

Figure S2. (A) Classification of 93 differential compounds in rice root exudates. (B) Venn's analysis of root exudates. CK: control group; Low: 2 mg kg^{-1}; High: 10 mg kg^{-1}.

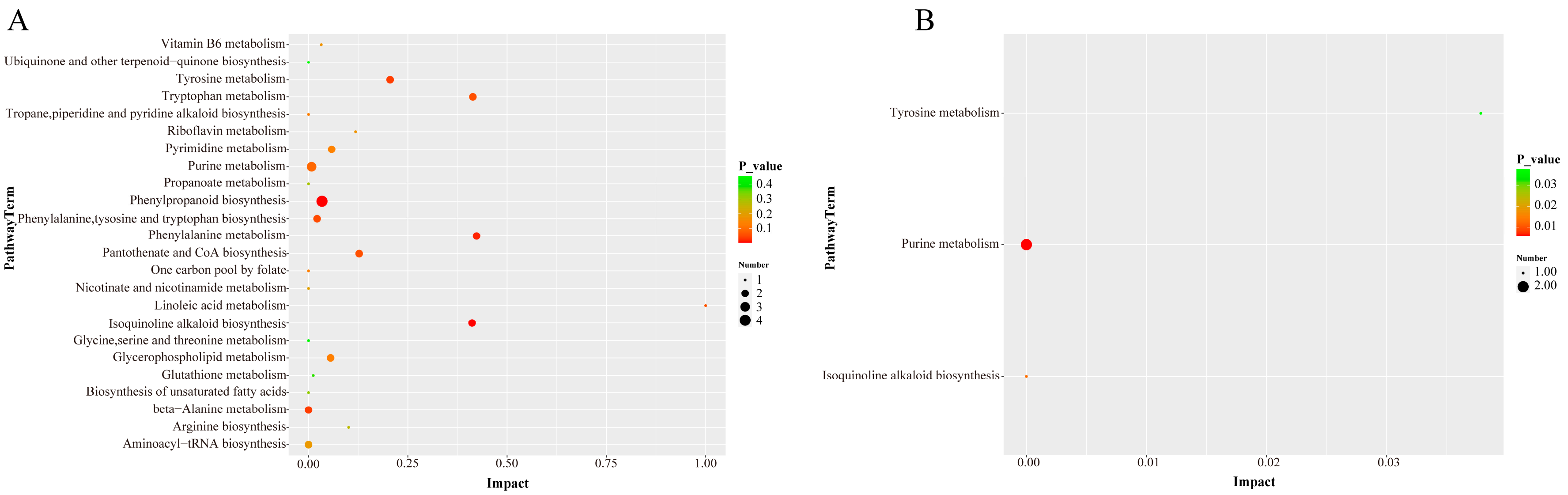

Figure S3. Metabolic pathway analysis of root exudates with significant differences in (A) low-treated (Low) and (B) high-treated (High) groups compared to control (CK) group.

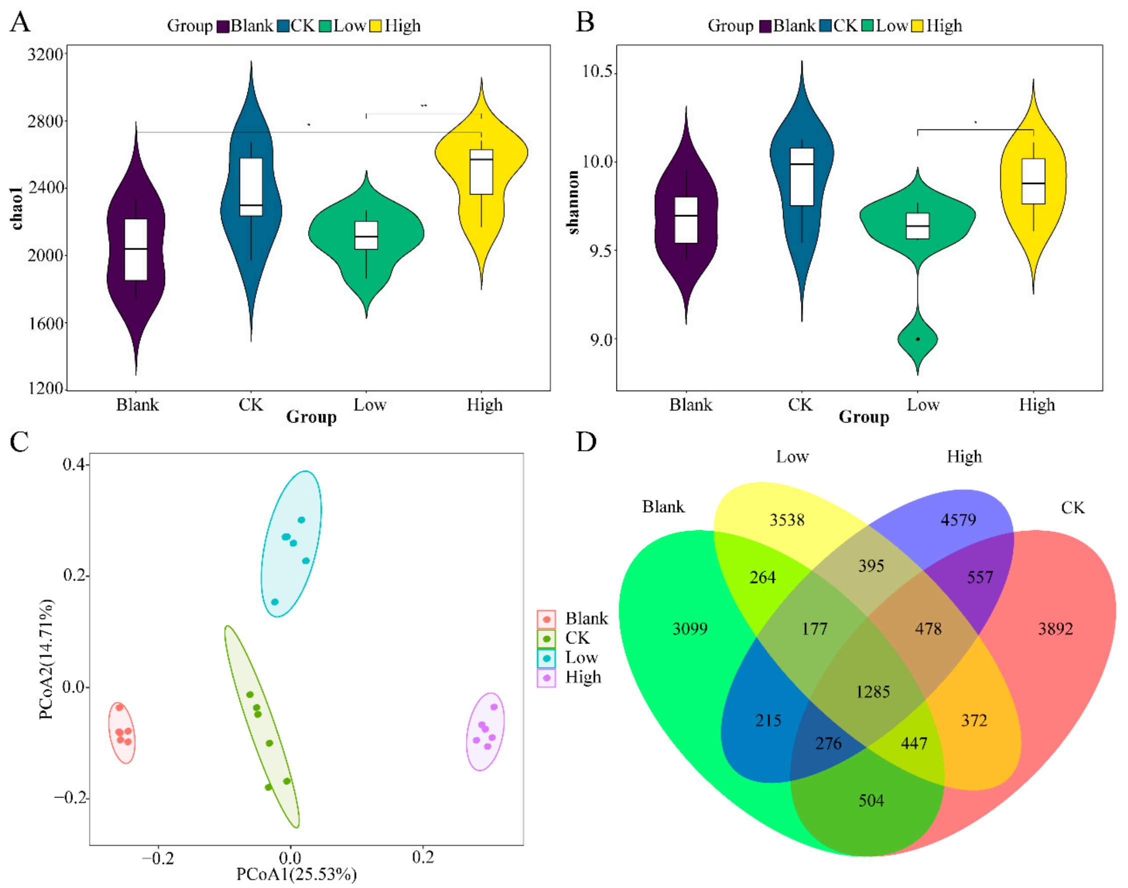

Figure S4. (A) Chao1 index (Index of mycorrhizal abundance); (B) Shannon index (Index of mycorrhizal diversity); (C) principal coordinate analysis (PCoA); and (D) Venn's analysis in rhizosphere soils. Blank: blank group; CK: control group; Low: 2 mg kg^{-1}; High: 10 mg kg^{-1}.

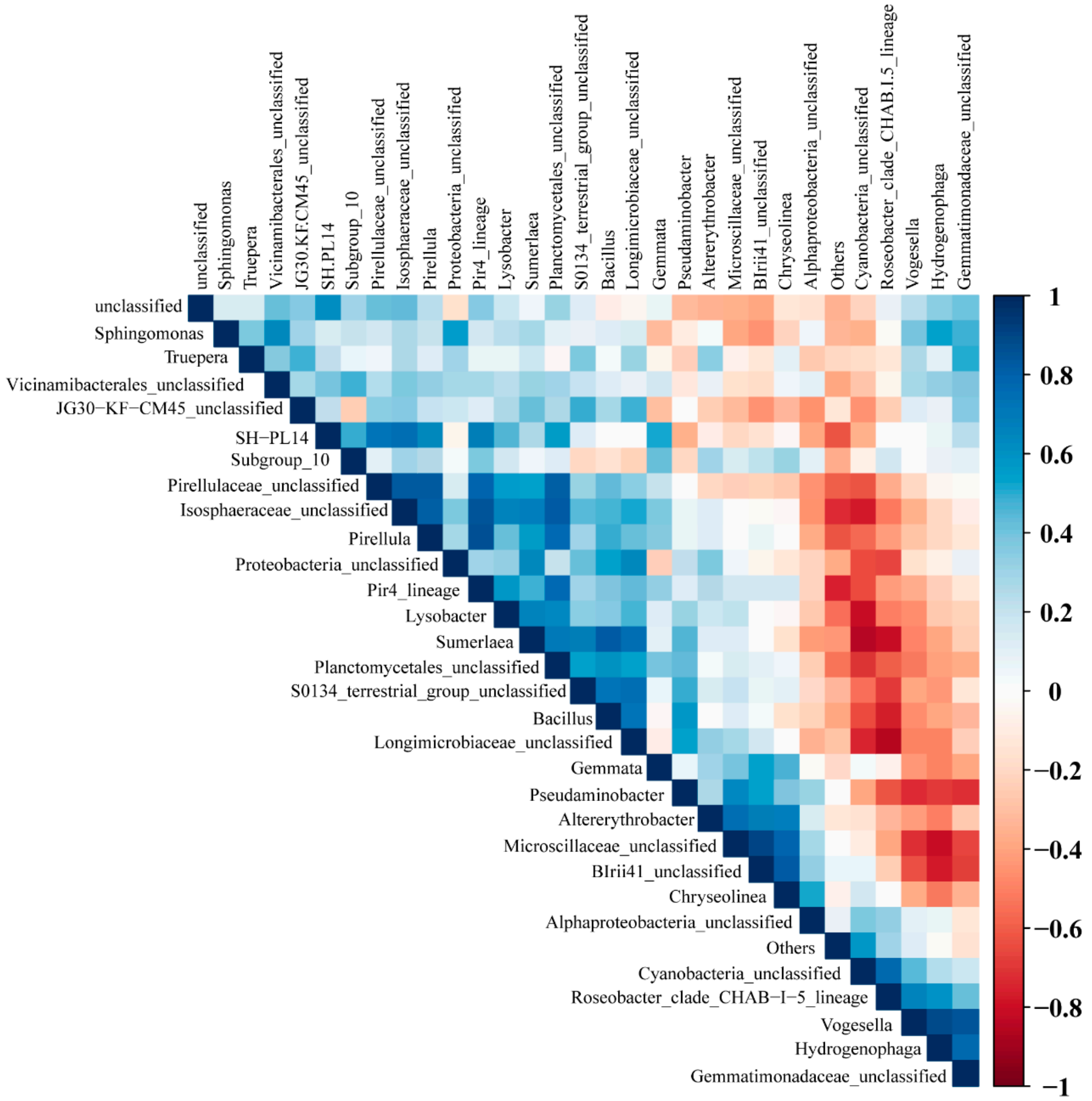

Figure S5. Hierarchical clustering analysis of microbial communities at genus level: correlation matrix of the Top 30 most abundant genera.

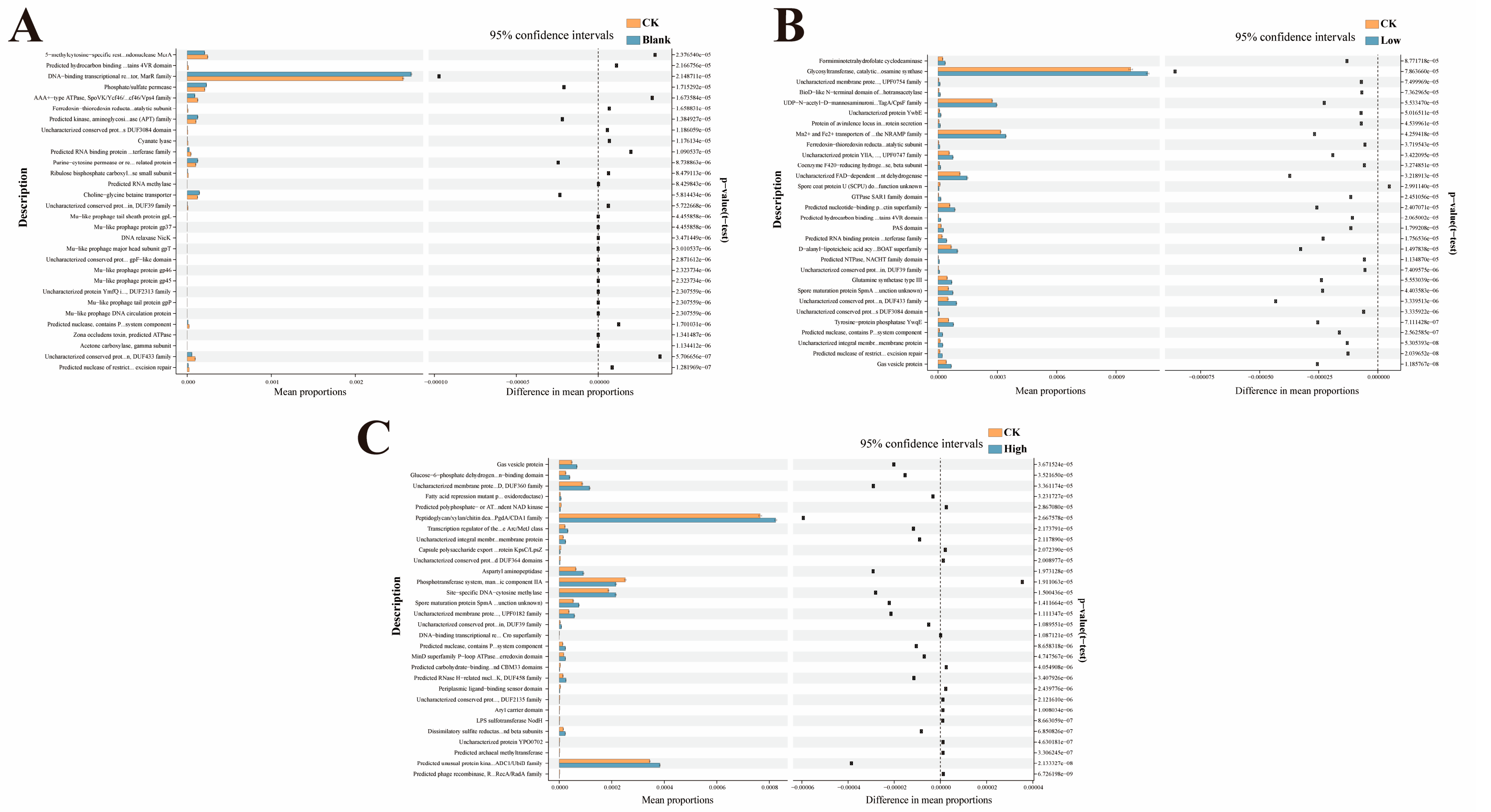

Figure S6. Statistical Analysis of Metagenomic Profiles (STAMP) ($P < 0.05$). （A）CK/ Blank; （B）CK/ Low; （C）CK/ High. The significant difference function between the two groups was obtained by this analysis.
